# A conceptual health economic modelling framework to assess the cost-effectiveness of molecular target–driven treatment regimens in oncology

**DOI:** 10.2340/1651-226X.2026.45088

**Published:** 2026-02-19

**Authors:** Oskar Frisell, Eline Aas, Pia Sofie Henkel, Gro Live Fagereng, Kjetil Taskén, Ebba Hallersjö Hult, Peter Lindgren, Katarina Steen Carlsson

**Affiliations:** a The Swedish Institute for Health Economics (IHE), Lund, Sweden; b Department of Learning, Informatics, Management and Ethics (LIME), Karolinska Institutet, Solna, Sweden; c Department of Health Management and Health Economics, University of Oslo, Oslo, Norway; d Division of Health Services, Norwegian Institute of Public Health, Oslo, Norway; e Institute for Cancer Research, Oslo University Hospital, Oslo, Norway; f Institute of Clinical Medicine, University of Oslo, Oslo, Norway; g Stockholm School of Economics, SIR, Stockholm, Sweden; h Lund University, Lund, Sweden

**Keywords:** Costs and cost analysis, medical oncology, models, economic, reimbursement mechanisms

## Abstract

**Background and purpose:**

Molecularly targeted cancer therapies challenge conventional health economic evaluation frameworks that are structured around tumour-specific indications, comparators, and trial designs. Existing models often rely on pooled estimates from heterogeneous early-phase evidence or single-indication analyses, creating uncertainty for reimbursement decision-makers. We propose a conceptual modelling framework that aligns cost-effectiveness analyses with the biological rationale of precision oncology, evaluating therapies according to shared molecular alterations across tumour types.

**Patient/material and methods:**

We examined the methodological limitations of conventional partitioned survival models (PSMs) commonly applied in oncology and evaluated their suitability for tumour-agnostic indications. Based on the collected literature, we developed a dynamic, modular PSM framework that integrates multiple tumour sites expressing a common biomarker. The framework supports pooled and tumour-specific analysis of cost-effectiveness and enables progressive disaggregation of subgroups as additional evidence becomes available.

**Results:**

The proposed modelling approach facilitates transparent synthesis of heterogeneous evidence across tumour types using epidemiologically informed weighting, while preserving the ability to estimate tumour-specific cost-effectiveness where data permit. It addresses key challenges in tumour-agnostic evaluation, including variation in standard of care, treatment effects, and resource use across cancer sites. The modular design promotes internal consistency, reduces duplication of analytical effort, and enables iterative re-assessment of both overall and subgroup-specific cost-effectiveness.

**Interpretation:**

A dynamic, weighted multi-site modelling framework represents a coherent and adaptable extension of current health-technology assessment-practice for tumour-agnostic therapies. By structuring evidence around molecular targets, the framework can improve transparency and robustness of cost-effectiveness estimates, thereby supporting more equitable and efficient reimbursement decisions in the context of precision oncology.

## Introduction

Precision cancer medicine and precision diagnostics have rapidly transformed the landscape of cancer care [1]. The sequencing of tumour genomes and the ability to identify actionable molecular alterations have allowed clinicians to prescribe molecularly targeted therapies that require specific genomic alterations to be effective. This shift challenges the traditional logic of treatment and drug discovery in oncology where treatments were developed, tested in trials, and reimbursed based on the tumour location. In recent years, therapies have been developed and granted marketing approval for any cancer carrying a given genomic variant [2–6]. These therapies have become symbolic of a new paradigm, where patients expressing a particular molecular biomarker or epitope independent of tumour location represent the main patient population, and tumour location instead serves to define patient subgroups.

This new type of ‘pan-cancer’ or ‘tumour-agnostic’ approach to the indication for a targeted therapy implies a shift in the definition of an indication from being for a specific form of cancer to the indication being a genomic alteration, which may appear in various tumour types expressing a resulting variant of relevance. For these types of indications, health economic evaluation models need to switch from a framework based on one tumour, one treatment, one comparator and one value assessment to a new framework of one genomic alteration, one treatment, numerous tumour types and numerous comparators.

A review of methods for economic evaluation of tumour-agnostic therapies identified that key challenges stem from non-randomised, single-arm basket trials with small and heterogeneous patient populations with limited long-term outcome data. This lack of robust evidence on effectiveness and relative effectiveness of therapies leads to uncertainty in both clinical and cost-effectiveness estimates. An incomplete understanding of the prognostic effects of biomarkers also makes it difficult to construct and define appropriate biomarkers. Suggested solutions to the challenges with clinical evidence include greater use of real-world data to define counterfactuals, increased statistical power, and making reasonable assumptions on longer term effects such as long-term health outcomes [7]. Adaptive, life-cycle health-technology assessment (HTA) frameworks such as managed entry agreements and ongoing re-evaluation of tumour-agnostic therapies are also called for in order to keep up with evidence generation and ensure sound health system resource allocation [7].

Model-based economic evaluations are commonly used to inform reimbursement decisions when survival benefits extend beyond observed trial follow-up. Molecular targets may be present across multiple tumour types with varying prevalence and tumour-specific standards of care, leading to heterogeneity in comparators, relative effectiveness, and adverse event profiles. The impact of targeted therapies on survival and health-related quality of life (HRQoL) further depends on the accuracy of diagnostic strategies used to identify biomarker-positive populations.

These sources of heterogeneity challenge the suitability of conventional tumour-specific cost-effectiveness models, which may inadequately capture variation across indications [9]. As a result, decision-makers face substantial uncertainty when determining whether and under what conditions targeted therapies should be reimbursed.

## Previous studies on evaluation of tumour-agnostic therapies

The literature on tumour-agnostic cost-effectiveness evaluations identified in an exploratory review in MEDLINE collectively points towards the value of a modular, weighted modelling framework rather than a single static approach. Early economic evaluations of, for example, larotrectinib and entrectinib adopt pragmatic pooled-effect models under substantial evidentiary constraints with tumour-specific comparators and weighting by tumour prevalence to enable assessment of therapy cost- effectiveness under limited evidence from single-arm trials [10–14].

Methodological work comparing alternative counterfactual constructions highlights that no single comparator strategy is sufficient and that triangulation or weighting across approaches may be necessary to characterise uncertainty when relative effectiveness cannot be directly observed due to heterogeneity in standard of care across tumour sites [9, 15]. More recent analyses challenge the assumption of homogeneous effects by modelling tumour-specific outcomes, using external controls to generate predicted tumour-level health outcomes and exploratory cost-effectiveness estimates, alongside aggregate value summaries [16, 17]. Together, the evidence from these studies supports an implicit progression, from pooled estimation towards selective tumour-level breakout only when evidence supports robust tumour-specific inference, while retaining weighted aggregation to reflect overall value across the eligible population [7, 18, 19]. A dynamic framework that combines early pooled evidence, modular tumour-specific components, and epidemiologically weighted costs, health effects and cost per quality-adjusted life years (QALY) offer a coherent and defensible alternative for evaluating tumour-agnostic therapies that can accommodate reanalysis as new evidence emerges.

This article presents a conceptual health economic modelling framework intended to support consistent, transparent, and biologically relevant assessments of cost-effectiveness for molecularly targeted oncology therapies. The framework aims to handle challenges with data availability and currently used research design of modern precision oncology, in which the same mutation can drive disease in diverse clinical contexts. The framework will translate into a decision analytic model to evaluate the cost-effectiveness of the PRIME-ROSE consortium Drug Repurposing Protocol (DRUP)-like clinical trials that use a combined umbrella-basket design where efficacy of drugs targeted to specific molecular alterations or biomarkers are investigated in parallel in different tumour types [20, 21].

## Partitioned survival models and cost-effectiveness analysis

Cost-effectiveness analysis (CEA) in late-stage oncology commonly employs partitioned survival models (PSMs), which remain the dominant modelling framework for evaluating new cancer therapies [22–24]. PSMs are particularly well suited to oncology because they rely directly on time-to-event outcomes routinely reported in clinical trials, most notably overall survival (OS) and progression-free survival (PFS).

In a typical PSM-based CEA, the target population, intervention, and comparator are defined based on phase III randomised controlled trial evidence. Published OS and PFS Kaplan–Meier curves are reconstructed, and parametric survival functions are fitted and extrapolated beyond trial follow-up to a sufficiently long, often lifetime, time horizon [22, 24]. Candidate distributions are assessed using statistical goodness-of-fit, visual inspection, and clinical plausibility, including external validation against registry data or expert opinion [22, 25]. Long-term survival projections may be adjusted for background mortality to ensure consistency with general population life expectancy.

PSMs usually comprise three mutually exclusive health states: progression-free disease, progressed disease, and death. State occupancy at each time point (t) is derived directly from the survival functions, with the proportion of progression-free defined by PFS(t), the proportion of dead by 1 – OS(t), and the proportion of progressed disease by OS(t) – PFS(t). Time spent in each state corresponds to the area under the respective survival curves. Because transitions between states are not explicitly modelled, PSMs can be implemented without individual patient-level data and align closely with reported trial endpoints [22, 24].

Health state-specific costs and utilities are assigned to estimate total discounted costs and QALYs. Costs typically include drug acquisition and administration, adverse event management, and end-of-life care, with the cost categories depending on the analytic perspective [8, 26, 27]. The key outcome metric is the incremental cost-effectiveness ratio (ICER) which describes the cost to gain one more QALY compared to, for example, SoC, which is compared against a context-specific willingness-to-pay threshold [28]. Parameter- and structural uncertainty are assessed using deterministic and probabilistic sensitivity analyses [8, 22, 24].

PSMs offer several advantages, including conceptual simplicity, transparency, and close alignment with regulatory trial evidence [22, 24]. However, they also have well-recognised limitations. The absence of explicit state transitions restricts their ability to capture complex disease pathways, treatment sequencing, or post-progression heterogeneity. In addition, independent extrapolation of OS and PFS makes results sensitive to parametric assumptions and may bias long-term survival and QALY estimates, particularly in heterogeneous or tumour-agnostic populations [15, 16, 22].

## Suggested new approach

### Added features

**Figure 1:**
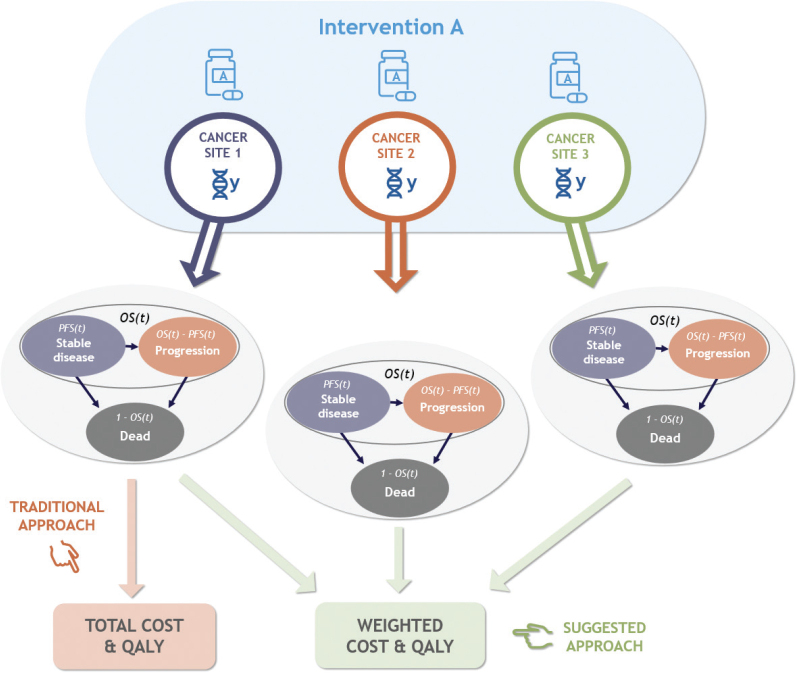
Model schematic: a comparison between a traditional PSM framework and the proposed weighted framework.

Building on existing evidence [9, 10], we propose an extension of the conventional framework that integrates weighted evidence across multiple tumour sites within a single, adaptive model structure (Figure 1). Clinical and economic data from tumour-specific indications are combined to estimate an aggregated ICER that reflects the average value of the therapy across the biomarker-defined population.

Weighting can theoretically be applied at different stages of the analysis, including individual cost components or health outcomes within tumour-specific submodels. However, because ratios cannot be meaningfully averaged, the level at which weighting is applied has important implications for the resulting aggregated ICER. Applying weights at highly granular levels is unlikely to support decision-making and may reduce transparency, particularly when cost structures and comparators differ across tumour types [22].

To ensure internal consistency and interpretability, we recommend applying weights to total or incremental costs and QALYs, from which a single aggregated ICER is derived. Weights should reflect the relative contribution of each tumour type to the overall biomarker-positive population, consistent with principles used for pooling clinical effectiveness evidence.

To address the limitations of static, single-indication models, we further propose that the framework allows for gradual disaggregation of tumour subgroups as evidence accumulates and uncertainty around subgroup-specific effects diminishes. This would entail a modular PSM design in which each tumour site or biomarker-defined subgroup can be evaluated both individually and as part of the aggregated model. As the evidence base for a given subgroup matures, that subgroup can be ‘broken out’ from the aggregate population to run its own dedicated submodel while remaining embedded within the overarching framework [7, 18]. Each submodel would incorporate the most relevant subgroup-specific inputs, such as OS and PFS data, health-state utilities, and cost parameters while maintaining consistency in structural assumptions, modelling conventions, and methodological choices across the full system (Figure 1). The approach with a fully aggregated analysis may lead to both over- and underestimation of the true value of molecularly targeted therapies when heterogeneity in clinical benefit or cost structures between tumour types is substantial.

A weighted, modular approach would preserve the interpretability of the aggregated analysis while providing greater granularity for subpopulations where sufficient data exist. This approach should give decision-makers a clearer understanding of both the average, overall, as well as the individual cost-effectiveness profiles across tumour sites. If a particular subgroup indicates that treatment with a specific therapy would not be cost-effective, this may be counterbalanced by other subgroups where the therapy demonstrates a more favourable cost-effectiveness profile. Importantly, this structure allows for transparent assessments of trade-offs between indications while maintaining a single evaluative framework, thereby supporting informed decisions on implementation or reimbursement of tumour-agnostic treatments.

Implementing this dynamic approach requires flexibility in technical design and modularity in programming. The proposed model should be constructed as a modular system, that can be reused and extended as new data emerge. This avoids the need to rebuild separate models for each re-analysis or indication expansion. Moreover, it should enhance internal validity over time: as modules are reused and updated prior iterations remain validated and consistent.

### Applications of PSM and new framework

Two central challenges arise in operationalising this framework. The first concerns timing of re-analysis, that is, determining when the accumulation of new evidence warrants updating the model and re-estimating cost-effectiveness. Frequent re-analyses may impose substantial analytical burden on decision-makers and consume unnecessary resources, whereas infrequent updates of cost-effectiveness estimate risk delaying access or sub-optimal use of health care resources. Evidence-based thresholds or pre-specified triggers for re-analysis, such as the availability of new subgroup-specific survival data or real-world evidence exceeding a defined sample size, could help manage this balance [7].

The second challenge relates to the collection and integration of real-world effectiveness data. For molecularly targeted therapies with tumour-agnostic indications, real-world evidence is likely essential to refine and validate subgroup-specific estimates of clinical effectiveness, especially when clinical trial data are limited or heterogeneous. However, such data collection can be complex as there may be differences in baseline prognosis, response rates, reporting of molecular profiling results and standard of care across tumour types. Furthermore, complexities arise by the variability in diagnostic testing and treatment sequencing in clinical practice. Additionally, variations in post-progression treatment paradigms and resource use complicate the estimation of relative effectiveness and incremental cost.

Aggregating all tumour sites into a single estimate of OS and PFS as well as costs, both for the intervention and the comparator(s), implicitly assumes homogeneity in treatment effect, disease trajectory, and healthcare resource use across cancers, which rarely holds true in practice. For example, the same genomic alteration may have a different prognosis or treatment response depending on the localisation of the tumour it is expressed in and the relevant standard of care. Ignoring such heterogeneity risks biasing the estimated ICER either upward or downward, depending on the relative distribution of favourable and unfavourable subgroups.

Despite these practical challenges, the proposed dynamic PSM framework offers several conceptual and methodological advantages. It promotes continuity between assessments, avoiding duplication of work while enabling evidence-based evolution of the model over time. It facilitates transparency by explicitly demonstrating how incremental data alter the cost-effectiveness of individual subgroups and overall assessments of molecularly targeted treatments [29]. It also potentially allows policymakers to visualise both overall and indication-specific value for money, strengthening the relevance of subsequent decisions. For therapies with wide-ranging or tumour-agnostic indications, this approach may support more equitable access by ensuring that clinically effective treatments remain available to patients, even if certain subgroups are less cost-effective in isolation. Conversely, this dynamic framework would also allow policy makers to identify subgroups for which treatment is cost-effective, even if the aggregated analysis suggests that using the evaluated therapy would not be cost-effective.

Sensitivity analyses such as deterministic one-way analyses (DSA) and probabilistic sensitivity analysis (PSA), where the latter is a method to assess the overall uncertainty of the model outcomes and inform subsequent decisions, can be conducted using standard approaches [8]. The key distinction relative to tumour-specific models lies not in the number of parameters per se, but in how parameters are structured and varied, particularly with respect to shared versus tumour-specific sources of uncertainty. Parameters representing common assumptions should be varied jointly across tumour sites, whereas tumour-specific parameters may be explored independently. This allows uncertainty to be examined both at the aggregate level and within individual tumour subgroups, supporting transparent interpretation of the drivers of decision uncertainty. One dimension of uncertainty not captured through PSAs or DSAs relates to structural assumptions embedded in the model design. For example, inadequate representation of the care pathway, or omission of relevant adverse events, health effects, or costs may influence model outcomes and can be, for example, examined through alternative structural specifications or scenario analyses.

In summary, a dynamic partitioned survival modelling framework that can evolve as evidence is generated could represent a logical extension of current HTA practice for molecularly targeted therapies in oncology. It would maintain the strengths of the traditional PSM structure while addressing the growing complexity of cancer treatment and the need for ongoing reassessment of value as real-world evidence emerges. As genomic-driven and tumour-agnostic therapies continue to expand, adopting such a flexible modelling paradigm will likely be essential for generating meaningful cost-effectiveness estimates to support implementation and reimbursement decisions for these therapies.

## Discussion

Shifting the perspective of health economic modelling and cost-effectiveness evaluation of molecularly targeted therapies in oncology, from a tumour-oriented to a mutation-oriented analysis, has the potential to reduce the uncertainty around whether these therapies provide good value for money.

The PRIMEROSE consortium was established to address evidence gaps in the clinical and economic evaluation of repurposing cancer drugs beyond their original tumour indications. One aim for the consortium is to develop and test methodological frameworks for assessing the value of molecularly guided therapies across tumour types [20]. The framework proposed in this paper will inform the development of a PRIMEROSE model and serve as a proof of concept for a modular and dynamic approach that supports re-evaluation as the evidence base evolves.

Alternative modelling approaches could also be applied within a similar tumour-agnostic paradigm. Discrete event simulation may ultimately be better suited to capture complex disease trajectories as it does not explicitly model each pathway [30], but such models currently require extensive data that may not yet be widely available. As tumour-agnostic therapies and comprehensive genomic profiling become more common, future data availability may warrant re-evaluation of the reliance on PSMs in this setting.

More conventional approaches, including Markov cohort models or microsimulation [31], may also be adapted to incorporate weighted subgroup evidence on cost-effectiveness using the same underlying rationale proposed here and represent important areas for future methodological research.

Current practice typically relies on separate economic models for each tumour site, resulting in methodological heterogeneity and potential duplication of effort. Integrating evidence across tumour sites into a unified modelling framework can improve internal consistency and enhance the transparency and reproducibility of economic evaluations for tumour-agnostic therapies.

A weighted PSM extends the conventional single-indication oncology model to the tumour-agnostic context by enabling joint estimation of overall and site-specific cost-effectiveness across cancers sharing a common biomarker. Its modular and nested structure can support efficient incorporation of new evidence and may facilitate re-analysis as data accumulate, allowing clinically meaningful subgroups to be evaluated with relatively limited additional modelling effort.

A key challenge for HTA bodies is to define transparent criteria for when accumulated evidence warrants re-analysis or subgroup delineation. Prospectively specified thresholds would balance methodological rigour with the need for timely reassessment, reduce ad hoc re-evaluations, and provide clearer expectations for manufacturers generating post-authorisation evidence.

The principal advantage of a harmonised modelling approach is its ability to capture heterogeneity in treatment effects, costs, comparators, and disease trajectories across tumour sites within a single analytical framework. This enables decision-makers to assess where tumour-agnostic therapies deliver the greatest incremental value and supports equitable, evidence-based resource allocation. A shared model structure also promotes methodological standardisation, reproducibility, and validation across re-analyses.

Recent work on multi-use disease models supports the use of dynamic economic modelling frameworks that are designed to evolve as evidence accumulates, rather than relying on static, single-use models developed for individual decisions [29]. These principles are relevant for tumour-agnostic oncology, where early evidence will need to be pooled across tumour sites and can then, if relevant, progressively be disaggregated as data matures. This approach enables reassessment of value while maintaining transparency, consistency, and methodological rigour across the technology life cycle. The dynamic nature of the PSM methodology and the modular approach suggested allows for a copy-paste expansion of the core model framework, where a new PSM module is easily incorporated as a stand-alone component and easily connected to the weighting convention.

Assuming an HTA perspective, the practical implementation of such a framework would benefit from clearly defined requirements for re-assessment. This could, for example, build on the Joint Clinical Assessment (JCA) process within the EU by requiring manufacturers and/or health care providers to collect, report and re-analyse evidence on relative treatment effects in tumour subgroups as a collaborative effort. Subsequently, updates on recommendations and re-assessments of these therapies could be performed in a unison and well-informed manner. Alternatively, such re-analyses could be leveraged through joint European initiatives such as the European Health Data Space (EHDS), facilitating data sharing and collaborative evidence generation. Introducing these obligations as conditional elements of marketing authorisation could potentially strengthen the link between HTA-body approval, data collection, and economic re-evaluation over time.

Adopting a weighted, multi-site model may involve important social and ethical trade-offs. Pooling evidence across tumour sites inherently needs to balance considerations on efficiency and equity. On one hand, weighted analyses can allow subgroups with favourable cost-effectiveness profiles to offset those with less favourable ones, thereby broadening access to clinically effective treatments. On the other hand, this may lead to less efficient resource allocation, as scarce healthcare resources are diverted towards subgroups that may not independently meet cost-effectiveness thresholds. This could lead to a reduction in overall health output in the health care system. The desire to maximise aggregate health benefits and ensuring equitable access could be a fundamental policy challenge for tumour-agnostic evaluations. By providing disaggregated and overall cost-effectiveness results, our proposed modelling framework enables a transparent visualisation and consideration of the potential trade-offs and equity considerations for molecularly targeted therapies in cancer care.

Decision- and policymakers are likely to prioritise patient subgroups with the most favourable cost-effectiveness profiles and, depending on perspective, this may be an appropriate approach to resource allocation. Similarly, pharmaceutical manufacturers have strong incentives to seek marketing authorisation in populations where commercial returns are expected to be greatest. While the proposed framework does not resolve these strategic considerations, it provides a methodological basis for evaluating tumour-agnostic therapies in a manner that is more consistent with their underlying clinical rationale. Specifically, it conceptualises tumour type as a subgroup within a biomarker-defined population, rather than treating the molecular target as a subgroup within a single tumour indication.

## Conclusion

A weighted multi-tumour-site modelling framework based on a modular structure offers a credible and transparent approach to the economic evaluation of tumour-agnostic therapies. By enabling structured evidence synthesis across tumour sites, it supports CEAs that reflect the evolving realities of precision oncology and facilitates both efficient and equitable decision-making. Importantly, its successful implementation will depend on clear regulatory expectations, robust data infrastructure, and sustained dialogue on the ethical limits of pooled economic evaluation. Ultimately this will lead to the alignment of tumour-agnostic innovation in oncology with a fair, adaptive, and forward-looking framework for assessing cost-effectiveness and value of these technologies.

## Data Availability

No data was processed in this work.
